# Sex Differences in Blood Accumulation of Neurodegenerative-Related Proteins and Antioxidant Responses to Regular Physical Exercise

**DOI:** 10.1007/s12031-024-02278-4

**Published:** 2024-11-05

**Authors:** Elisa Chelucci, Giorgia Scarfò, Rebecca Piccarducci, Antonio Rizza, Jonathan Fusi, Francesco Epifani, Sara Carpi, Beatrice Polini, Laura Betti, Barbara Costa, Sabrina Taliani, Vito Cela, Paolo Artini, Simona Daniele, Claudia Martini, Ferdinando Franzoni

**Affiliations:** 1 Department of Pharmacy, University of Pisa, Pisa, Italy; 2 Division of General Medicine, Department of Clinical and Experimental Medicine, University of Pisa, Pisa, Italy; 3 Interventional Cardiology Division, Gaetano Pasquinucci Heart Hospital, Fondazione Toscana Gabriele Monasterio, Massa, Italy; 4 Department of Juridical and Economic Sciences, Pegaso Telematic University, Fanfani Diagnostics and Health, Florence, Italy; 5 Department of Health Sciences, University “Magna Graecia” of Catanzaro, Catanzaro, Italy; 6 National Enterprise for nanoScience and nanoTechnology (NEST), Istituto Nanoscienze-CNR and Scuola Normale Superiore, Pisa, Italy; 7 Department of Pathology, University of Pisa, Pisa, Italy; 8 Division of Gynecology and Obstetrics, Azienda Ospedaliero Universitaria Pisana, Pisa, Italy; 9 Department of Clinical and Experimental Medicine, University of Pisa, Pisa, Italy

**Keywords:** Neurodegenerative diseases, Physical activity, Biological sex, Antioxidant capability, Epigenetic mechanisms

## Abstract

Physical activity has been demonstrated to improve cognitive function, thereby preventing/slowing neurodegenerative diseases (NDs). Biological responses to physical activity and vulnerabilities to NDs are emerging to be gender-related. Herein, known ND-associated markers (β-amyloid, tau, α-synuclein), main sex steroid hormones, antioxidant responses, and key gene transcription modulators were evaluated in the blood of physically active and sedentary women and men. In our hands, females presented higher basal erythrocytes β-amyloid and α-synuclein amounts than males. Regular physical activity was able to significantly reduce the erythrocyte content of β-amyloid in females and the tau levels in males, suggesting that these differences may be mediated by organizational actions of sex steroid hormones during development. Furthermore, despite a comparable plasma antioxidant capability (AOC) between males and females, in the latter group, physical activity significantly enhances AOC versus peroxynitrite radicals only. Finally, regular physical activity modulated the levels of transcription factor Nrf2 in erythrocytes, as well as the plasma concentration of the microRNA miR-195 and miR-153, suggesting the promotion of antioxidant/autophagic processes associated with ND-related proteins. Overall, these results could shed light on how cerebral adaptations to physical activity differ between males and females, especially with regard to blood accumulation of ND proteins and mechanisms of antioxidant responses to regular exercise.

**Supplementary Information:**

The online version contains supplementary material available at 10.1007/s12031-024-02278-4.

## Introduction

Neurodegenerative diseases (NDs) are multifactorial pathologies characterized by the progressive impairment of the neuronal structure and function (Chen et al. 2016; Scarfò et al. 2021). Among NDs, dementia affects almost 47 million people, and this number is expected to reach 74.7 million by the year 2030 with an estimated total worldwide economic burden of 2 trillion dollars (Ijaopo 2017).

Alzheimer’s disease (AD) represents the most common type of dementia. Typical extracellular plaques of β-amyloid 1–42 peptide (Aβ) accumulate in AD brains (Takahashi et al. 2017) and are deleterious to neuronal cells as they can impair neuronal membrane permeability triggering alteration in numerous intracellular pathways that cause cell dysfunction and apoptosis (Cheignon et al. 2018).

Although NDs affect both women and men, the female gender is considered a risk factor for dementia development, showing a twofold greater risk at later ages (Pinares-Garcia et al. 2018; Rahman et al. 2019), faster progression of brain atrophy (1–2.5% per year) (Ardekani et al. 2016; Hua et al. 2010), and worse AD-related pathology (Barnes et al. 2005; Gao et al. 1998) than men, although the influence of gender on AD onset is complex and multifactorial (Barha & Liu-Ambrose 2018; Congdon 2018). In contrast, men are more predisposed to Parkinson’s disease (PD) and amyotrophic lateral sclerosis (Cortes & De Miguel 2022) development.

In the context of dementia and its related influencing factors, gender differences can influence biological as well as psychological and behavioral aspects. Particularly, genetic, hormonal, and social profiles are diversified in women and men; indeed, despite the overlapping of numerous factors, sex differences specifically affect pathways and mechanisms involved in brain health in a sex-specific manner (Cortes & De Miguel 2022).

Besides genetic factors, it is well-known that regular physical activity delays the onset of dementia and reduces brain degeneration by improving blood markers of brain health and psychological flags associated with cognitive impairment (Jia et al. 2019). In particular, moderate exercise activity can improve antioxidant defenses and autophagy mechanisms in brain cells preventing the accumulation of oligomeric proteins, including Aβ (Piccarducci et al. 2021), thereby reducing the risk of developing AD by 45% (Hamer & Chida 2009), while individuals with a sedentary lifestyle are 53% more likely to develop AD than those who engaged in more active lives (Buchman et al. 2012). Considering the numerous emerging data from meta-analyses concerning this topic, a huge variability of results regarding the cognitive adaptations to exercise is coming out (Aghjayan et al. 2021; Northey et al. 2018; Roig et al. 2013; Sanders et al. 2019; Xue et al. 2019), mainly because of different sample management, dementia substrates, protocols, and setups, especially due to the biological and gender differences of participants involved in the analyzed studies (Carone et al. 2014; Groot et al. 2016; Ludyga et al. 2020). Nevertheless, some available studies suggest the potential association of sex differences with exercise and physical activity. For instance, the release of neurotrophic factors, cognitive functions, and brain homeostasis in general are differentially modulated depending on sex differences (Barha et al. 2019; Barha & Liu-Ambrose 2018; Cortes & De Miguel 2022). Despite the many studies investigating the impact of exercise on cognitive and brain function in humans, precious little is known about the biological mechanisms underlying these effects, especially as concerns sex differences (Barha & Liu-Ambrose 2018).

Therefore, the aim of the present study is to investigate putative sex-specific differences in the accumulation of ND-related proteins and antioxidant responses, as well as intracellular mechanisms that influence adaptations to regular physical exercise in a population of premenopausal women as compared to age-matched men.

## Materials and Methods

### Healthy Subjects: Enrollment, Setup of Clinical Parameters, and Classification

Healthy subjects (120) were enrolled at the Department of Clinical and Experimental Medicine of the University of Pisa (Sports Medicine Unit), with the following inclusion criteria: (i) diastolic/systolic arterial blood pressure minor than 90–140 mmHg; (ii) plasma triglycerides minor than 150 mg/mL; and (iii) total plasma cholesterol and HDL cholesterol ranging from 120 to 220 mg/mL and from 26 to 75 mg/mL, respectively. Moreover, the present study included women with a regular physiological menstrual cycle. Women under hormonal medications or in menopause were excluded from the study. Enrolled subjects followed the Mediterranean diet. The exclusion criteria were as follows: (i) body mass index higher (BMI) than 30 kg/m^2^; (ii) being a smoker; (iii) pharmacological treatment or supplementation with antioxidant properties; (iii) cardiovascular disease; (iv) familiar AD cases; (v) be carriers of apolipoprotein E e4 isoform, identified by restriction fragment length polymorphism (RFLP), using genomic DNA extracted from blood of healthy subjects (Daniele et al. 2018a, b, c, d). The final enrollment was based on clinical history, physical examination, and basal and stress electrocardiography (Daniele et al. 2018a, b, c, d; Piccarducci et al. 2019, 2021). The study was approved by the Ethics Committee of the Great Northwest Area of Tuscany (271/2014 to F.F. and protocol code 35/105, approved on 13 June 2019 to V.C.) in accordance with the Declaration of Helsinki. Each subject was enrolled in the study only after the acceptance of the informed consent. Fully informed consent was obtained from each subject entering the study (Piccarducci et al. 2021). The in-house database (Piccarducci et al. 2021) was implemented for up to 120 subjects whose samples were used to analyze further parameters.

The enrolled population was divided into physically active individuals (ATHL) versus age- and sex-matched sedentary (SED) subjects (controls). ATHL performed 150 min per week of moderate aerobic fitness, as established by the World Health Organization (WHO) (Wicker & Frick 2016), for at least 10 years. The intensity of physical exercise per attendee was estimated by the Borg Rating and Perceived Exertion (RPE) scale, which is a validated means to assess the subjective perception of effort. Specifically, the RPE scale consists of 15 numbers that range from a score of 6 to 20; 6 is ascribed to exercises performed without effort perception (low intensity) and 20 to maximal effort perception (high intensity) (Borg 1982; Piccarducci et al. 2021).

### Collection of Whole Blood, Plasma, and Red Blood Cells

The whole blood was collected in an EDTA-containing tube, at least 2 days later from the last exercise bout. The blood was centrifuged at 300 × *g* at 4 °C for 10 min to separate erythrocytes from plasma (Daniele et al. 2018a, b, c, d). The erythrocytes were further centrifuged at 1000 × *g* for 10 min and washed three times with phosphate buffer. Plasma and erythrocytes were stored in different aliquots until use.

### Determination of 17-b-Estradiol and Testosterone

17-b-Estradiol and testosterone were quantified in plasma following the manufacturer’s instructions by commercial immunoenzymatic assays (ab108667 and ab108666, Abcam). The hormonal levels were expressed as pg/ml.

### Evaluation of the Total Antioxidant Capability (AOC)

The plasma antioxidant capability (AOC) toward peroxyl radicals (ROO), hydroxyl radicals (OH), and peroxynitrite (OONO) was measured by the total oxyradical scavenging capacity (TOSC) assay a gas chromatographic technique determining the oxyradical scavenging ability of biological fluids, as previously described (Daniele et al. 2018a, b, c, d; Verratti et al. 2022). Particularly, the AOC of a molecule or biological liquid is quantified by its ability to inhibit ethylene formation compared with a control solution; the ethylene production was measured after the addition of plasma to a solution containing peroxyl (ROO), hydroxyl (OH), or peroxynitrite (OONO) radicals and 0.2 mM alpha-keto gamma-methylthiobutyric acid (KMBA). TOSC values (units/mL) were quantified from the following equation: TOSC = 100 − (∫SA/∫CA × 100), where ∫SA and ∫CA are the integrated areas calculated under the kinetic curve produced during the sample (∫SA) and control (∫CA) reactions (Verratti et al. 2022). A TOSC value of zero corresponds to a sample not presenting a scavenging capacity (Daniele et al. 2018a, b, c, d; Verratti et al. 2022).

### Quantification of ND-Related Protein Concentrations in Erythrocytes

Erythrocytes are of pivotal interest in considering oxidative stress, aging mechanisms, and ND-related protein accumulation (Kiko et al. 2012). In particular, the literature data suggest that red blood cells store Aβ (Kiko et al. 2012) and represent the major source of synuclein (Barbour et al. 2008). For these reasons, the quantification of α-syn, Aβ, and tau was assessed in erythrocytes and was measured by an enzyme-linked immunosorbent assay (ELISA), as already described (Daniele et al. 2018a, b, c, d; D’Antongiovanni et al. 2021; Piccarducci et al. 2019, 2021). Of note, specific antibodies against the Aβ 1–42 peptide were chosen, in order to detect the presence of peptide form that is implicated in fibrils formation and oligomerization process (Giacomelli et al. 2017; Sengupta et al. 2016). The plate was pre-coated with a specific antibody (α-syn, NBP2-15,365, Novus Biological; Aβ, 44,344, Invitrogen; tau, SC-32274, Santa Cruz) in poly-*L*-ornithine overnight at 4 °C. After three washes with 200 µL of PBS-T (PBS, containing 0.01% Tween 20), BSA 1% was added and incubated at 37 °C in order to block non-specific sites. Then, erythrocytes (40 µg/100 µL) were added to each well and incubated at 25 °C. Later, a primary antibody directed against the protein of interest (α-syn, SC-12767, Santa Cruz Biotechnology; Aβ, SC-28365, Santa Cruz Biotechnology; tau,ab109392, Abcam) was incubated at 25 °C for 2 h. Subsequently, following three washing with 200 µL of PBS-T, a horseradish peroxidase (HRP) secondary antibody (Sigma-Aldrich) was added and incubated at 37 °C. After the development of the colorimetric reaction by HRP substrate (3,3,5,5-tetramethylbenzidine, TMB, ThermoFisher), the absorbance was read at 450 nm (EnSight Multimode Plate Reader, PerkinElmer).

Finally, oligomeric α-syn was quantified in erythrocytes using aged solutions of the protein, as reported before (Daniele et al. 2018a, b, c, d; El‐Agnaf et al., 2006). The samples were added for 2 h on a plate pre-coated overnight with the mouse monoclonal α-syn 211 antibody (Santa Cruz, sc-12767). The oligomeric α-syn was identified using an α-syn biotinylated antibody (Daniele et al. 2018a, b, c, d; El‐Agnaf et al., 2006) and a streptavidin–horseradish peroxidase conjugate antibody (GE Healthcare). After three washes with PBS-T, 100 µL of TMB were added to each well, as reported above.

The standard curve was built by employing recombinant human α-syn (total or oligomeric), Aβ, or tau solution at different concentrations, and the relative concentration of each specific protein was calculated according to the standard curve obtained in each microplate (Daniele et al. 2018a, b, c, d; Daniele et al. 2018a, b, c, d; Piccarducci et al. 2019, 2021, 2022).

### Quantification of Nrf2 in Erythrocytes

The nuclear factor erythroid 2-related factor 2 (Nrf2) quantification was performed by an ELISA kit (Nrf2 Transcription Factor Assay Kit, colorimetric, Abcam, #ab207223), to quantify the Nrf2 active form^**14**^. Briefly, erythrocytes (10 µL, corresponding to 5–20 µg of total proteins, quantified 330 through Lowry assay) were diluted in the completed binding buffer and incubated for 1 h at 25 °C under shaking. Following, a primary antibody was incubated for 1 h at 25 °C, and after extensive washing, a secondary antibody was incubated for 1 h at 25 °C. After the incubation with the developing solution and following the stop solution addition, the absorbance was read at 450 nm (EnSight Multimode Plate Reader, PerkinElmer). The Nrf2 amount was calculated from Nrf2 activation absorbance and normalized to the absorbance of the total proteins in samples (µg/µL) (Piccarducci et al. 2021).

### Erythrocyte HDAC6

The histone deacetylase 6 (HDAC6) was measured in erythrocytes through a competitive ELISA kit (Human Histone Deacetylase 6 (HDAC6) Elisa Kit, Competitive ELISA, MyBioSource, #MBS7254230) (Piccarducci et al. 2021). Briefly, erythrocytes (100 µL diluted in PBS) were incubated for 1 h at 37 °C with a balance solution and conjugate. Then, the wells were washed three times. After the addition of the substrate solution and the following stop solution, thus blocking the enzyme–substrate reaction, the absorbance was read at 450 nm (EnSight Multimode Plate Reader, PerkinElmer) (Piccarducci et al. 2021).

### Levels of DNA Methyltransferase (DNMT)

The blood concentrations of DNMT1 and DNMT3A (Mizuno et al. 2001) were assessed by specific immunoenzymatic assays, following the manufacturer’s instructions (Cat#EKU03768-96 T and Biomatik Corporation, Ontario, Canada), as described by Daniele et al. (2018a, b, c, d). The standards or samples (100 µL) were added into the precoated (biotin-conjugated antibody) wells and incubated with HRP-streptavidin conjugate. Thereafter, TMB substrate solution (3,3,5,5-tetramethylbenzidine) was added. The reaction stopped and the absorbance was read at 450 nm. The data were expressed as pg/mg of total proteins.

### Analysis of the Expression of Circulating miRNA

miRNAs were isolated from plasma samples using a miRNeasy Serum/Plasma Mini Kit (Qiagen, Hilden, Germany). cDNA was obtained by a retro transcription reaction using the miRCURY LNA miRNA RT Kit (Qiagen, Hilden, Germany). Real-time PCR was assayed using a specific miRCURY LNA miRNA PCR Assay (Qiagen, Hilden, Germany), as previously reported(Piccarducci et al. 2021). The miRCURY Primer Assay specific for hsa-miR-195-5p (MIMAT0000461: 5′ UAGCAGCACAGAAAUAUUGGC), hsa-miR-153-3p (MIMAT0000439: 5′ UUGCAUAGUCACAAAAGUGAUC), and hsa-miR-93-5p (MIMAT0000093: 5′ CAAAGUGCUGUUCGUGCAGGUAG) was purchased from Qiagen (Hilden, Germany). The relative miRNA expression was calculated using the Ct method and normalized on miR-93-5p, employed as a plasmatic reference gene in the manufacturer’s handbook (Piccarducci et al. 2021).

### Statistical Analysis

The GraphPad Prism (GraphPad Software Inc., San Diego, CA, USA) was used for data analysis and graphical presentations. The data are presented as the mean ± SD or median, as indicated. Statistical analyses were performed by the unpaired *t*-test or Mann–Whitney for normal and non-normal distributed data, respectively.

To understand if the measured biochemical parameters could be influenced by hormonal levels or physical activity, correlations between parameters for each gender group were determined by simple linear regression analysis, using the StatView program (Abacus Concepts, Inc., SAS Institute, Cary, NC, USA). In particular, the nature of the correlation between the variables was plotted in a scatter diagram, and the simple correlation coefficient was reported as “*R*2”. The analyses will be considered significant with a 95% confidence interval (CI) from the mean and a *p*-value < 0.05.

## Results

### Descriptive Statistics

As shown in Table 1, age and body mass index (BMI) values did not differ significantly between females (ATHL and SED) and males (ATHL and SED).

**Table 1:** Descriptive statistics of selected parameters for females and males, distinguished in athletes (ATHL) and sedentary (SED) groups

Parameters	Female (media ± SD)		Male (media ± SD)	
	ATHL (media ± SD)	SED (media ± SD)	ATHL (media ± SD)	SED (media ± SD)
Age (years)	42.27 ± 9.79		42.30 ± 12.43	
	40.41 ± 8.27	44.10 ± 10.77	37.96 ± 12.28	44.68 ± 11.75
BMI (kg/m^2^)	21.00 ± 1.90		22.00 ± 1.60	
	20.80 ± 1.50	21.40 ± 1.20	21.50 ± 1.90	22.50 ± 1.20
Borg 15 (RPE)	9.22 ± 3.43		10.13 ± 3.92	
	13.32 ± 1.76^***^	6.78 ± 1.02	14.31 ± 1.57^§§§§^	6.94 ± 1.20
Estradiol (pg/ml)	58.30 ± 24.47^#^		48.49 ± 21.93	
	58.19 ± 21.65	58.37 ± 26.13	42.43 ± 12.97	53.12 ± 26.10
Testosterone (pg/ml)	28.73 ± 15.85^####^		540.17 ± 209.39	
	36.08 ± 14.84^**^	24.77 ± 15.11	673.01 ± 221.38^§§§§^	438.52 ± 129.20

All data are expressed as mean ± SD. Statistical analysis was performed by unpaired *t*-test between females (60 subjects) and males (60 subjects), female ATHL (30 subjects) and female SED (30 subjects), and between male ATHL (30 subjects) and male SED (30 subjects)

*BMI* body mass index, *Borg 15* Borg rating of perceived exertion scale

^#^*p* < 0.05, ^####^*p* < 0.0001, females vs males; ^**^p < 0.01, female ATHL vs female SED; ^§§§§^*p* < 0.0001 male ATHL vs male SED

As expected, the intensity of physical activity was significantly higher in the ATHL group (females and males) compared to the SED one (*p* < 0.0001). Obviously, the female group has shown significantly higher estradiol levels (*p* = 0.0224; Table 1) and lower testosterone (*p* < 0.0001; Table 1) in blood, compared to the male one.

Furthermore, ATHL exhibited significantly higher circulating testosterone levels than SED, independently from sex (female ATHL vs female SED, *p* = 0.0073; male ATHL vs male SED, *p* < 0.0001; Table 1), thus demonstrating that physical activity enhances testosterone levels in both females and males.

### Biochemical Parameters

All clinical and biochemical parameters investigated in females (ATHL and SED) and males (ATHL and SED) are reported in Tables 2, 3, and 1S.

**Table 2:** Selected clinical and biochemical parameters for females and males

Parameters	Female	Male
TOSC ROO (AOC)	15.42 ± 4.67	14.99 ± 3.64
TOSC OH (AOC)	5.86 ± 2.47	6.64 ± 2.64
TOSC ONOO (AOC)	17.48 ± 5.12	16.79 ± 4.86
Aβ (ng/mg protein)	12.69 ± 8.32^#^	9.84 ± 6.85
Tau (ng/mg protein)	8.09 ± 7.65	7.85 ± 7.42
α-syn (ng/mg protein)	71.09 ± 66.53^#^	48.90 ± 45.32
Oligomeric α-syn (ng/mg protein)	11.15 ± 5.60	10.38 ± 4.47
Nrf2 (Abs ratio Nrf2/Abs tot proteins (µg/µL)	15.85 ± 6.24	15.97 ± 6.27
miR-153 (relative expression)	0.009 ± 0.013	0.011 ± 0.014
miR-195 (relative expression)	0.017 ± 0.015	0.019 ± 0.017
HDAC6 (pg/mg protein)	23.16 ± 9.18	22.51 ± 9.51
DNMT1 (pg/mg protein)	403.58 ± 331.67	473.59 ± 272.48
DNMT3A (pg/mg protein)	166.46 ± 116.84	148.88 ± 94.85

All data are expressed as mean ± SD. Statistical analysis was performed by unpaired *t*-test between females (60 subjects) and males (60 subjects)

^#^*p* < 0.05, females vs males

*TOSC ROO* TOSC values vs peroxyl radicals, *TOSC OH* TOSC values vs hydroxyl radicals, *TOSC ONOO* TOSC values vs peroxynitrite radicals, *Aβ* beta-amyloid, *α-syn* alpha-synuclein, *Nrf2* nuclear factor erythroid 2-related factor 2, *miR* miRNA, *HDAC6* histone deacetylase 6, *DNMT1* DNA methyltransferase 1, *DNMT3A* DNA methyltransferase 3A

**Table 3:** Selected clinical and biochemical parameters for females and males, distinguished in ATHL and SED groups

	Female		Male	
Parameters	ATHL	SED	ATHL	SED
TOSC ROO (AOC)	16.46 ± 5.86	14.90 ± 3.94	16.42 ± 3.45^§§^	13.89 ± 3.43
TOSC OH (AOC)	6.27 ± 1.81	5.65 ± 2.74	8.28 ± 2.45^§§§§^	5.31 ± 2.00
TOSC ONOO (AOC)	21.65 ± 3.70^****^	15.40 ± 4.44	20.40 ± 4.26^§§§§^	14.02 ± 3.21
Aβ (ng/mg protein)	8.94 ± 4.33^**^	14.70 ± 9.26	9.62 ± 7.54	10.01 ± 6.38
Tau (ng/mg protein)	5.62 ± 2.50	9.41 ± 9.0	5.61 ± 4.25^§^	9.57 ± 8.82
α-syn (ng/mg protein)	79.46 ± 63.1	66.80 ± 68.6	45.57 ± 47.10	51.45 ± 44.46
Oligomeric α-syn (ng/mg protein)	10.44 ± 4.02	11.53 ± 5.3	9.67 ± 4.07	10.93 ± 4.74
Nrf2 (Abs ratio Nrf2/Abs tot proteins (µg/µL)	18.81 ± 6.25^**^	14.20 ± 5.67	19.72 ± 5.54^§§§§^	13.10 ± 5.24
miR-153 (relative expression)	0.006 ± 0.008	0.011 ± 0.014	0.005 ± 0.003^§§^	0.015 ± 0.017
miR-195 (relative expression)	0.024 ± 0.016^**^	0.013 ± 0.012	0.025 ± 0.021^§^	0.015 ± 0.013
HDAC6 (pg/mg protein)	16.45 ± 6.61^****^	26.77 ± 8.34	16.18 ± 6.57^§§§§^	27.50 ± 8.48
DNMT1 (pg/mg protein)	426.93 ± 421.0	391.00 ± 277	465.65 ± 300	480.04 ± 252.57
DNMT3A (pg/mg protein)	137.89 ± 85.78	182.68 ± 129.5	145.10 ± 131.9	151.55 ± 58.15

All data are expressed as mean ± SD. Statistical analysis was performed by unpaired *t*-test between female ATHL (30 subjects) and female SED (30 subjects); and between male ATHL (30 subjects) and male SED (30 subjects)

^*^p < 0.05, ^**^p < 0.01, ^****^p < 0.0001, female ATHL vs female SED; ^§^*p* < 0.5, ^§§^*p* < 0.01, ^§§§§^*p* < 0.0001, male ATHL vs male SED

*TOSC ROO* TOSC values vs peroxyl radicals, *TOSC OH* TOSC values vs hydroxyl radicals, *TOSC ONOO* TOSC values vs peroxynitrite radicals, *Aβ* beta-amyloid, *α-syn* alpha-synuclein, *Nrf2* nuclear factor erythroid 2-related factor 2, *miR* miRNA, *HDAC6* histone deacetylase 6, *DNMT1* DNA methyltransferase 1, *DNMT3A* DNA methyltransferase 3A

Plasma AOC was determined by the TOSC assay. TOSC values against peroxyl (*p* = 0.5775), hydroxyl radicals (*p* = 0.1025), and peroxynitrite (*p* = 0.4531) radicals did not differ significantly between female and male groups (Table 2).

As expected, ATHL exhibited significantly higher plasma antioxidant capacity than SED (TOSC vs peroxyl radicals, *p* = 0.0102; TOSC vs hydroxyl radicals, *p* < 0.0001; TOSC vs peroxynitrite radicals, *p* < 0.0001; Table 1S), especially considering the male group (male ATHL vs male SED, TOSC vs peroxyl radicals, *p* = 0.0065; TOSC vs hydroxyl radicals, *p* < 0.0001; TOSC vs peroxynitrite radicals, *p* < 0.0001; Table 3).

In contrast, in the female group, only plasma TOSC values against peroxynitrite radicals in ATHL were significantly elevated compared to SED (*p* < 0.0001; Table 3), whereas plasma TOSC values against peroxyl and hydroxyl radicals were comparable between the two groups (TOSC vs peroxyl radicals, *p* = 0.2371; TOSC vs hydroxyl radicals, *p* = 0.3774). These data confirm that physical exercise is able to enhance plasma antioxidant capabilities in human subjects and it was prevalent in males.

In addition, blood assessment of ND-related proteins was also considered in erythrocytes, which have been suggested as a good model for studying aging mechanisms and the accumulation of ND-related proteins (Barbour et al. 2008; Kiko et al. 2012).

Of note, the male group presented significantly lower levels of Aβ (*p* = 0.0430; Table 2) and α-syn (*p* = 0.0353; Table 2) compared to the female one. In general, no significant differences emerged between ATHL and SED (*p* = 0.1931; Table 1S) or between females and males (*p* = 0.4100; Table 2) considering the oligomeric form of α-syn. Similarly, oligomeric α-syn did not significantly differ between female ATHL and SED (*p* = 0.4800; Table 1S) and between male ATHL and SED (*p* = 0.2817; Table 3). Moreover, SED subjects presented significantly higher concentrations of Aβ (*p* = 0.0258; Table 1S) and tau (*p* = 0.0053; Table 1S) than ATHL. Also, erythrocytes of females ATHL exhibited lower levels of Aβ than the SED counterpart (*p* = 0.0093; Table 1S). Similarly, in the male group, ATHL showed lower erythrocyte levels of tau compared to SED (*p* = 0.0394; Table 3).

Moreover, Nrf2 was quantified in erythrocytes of all groups, as a regulator of antioxidant response and autophagic processes related to ND proteins (Daniele et al. 2018a, b, c, d).

Levels of Nrf2 expression in erythrocytes did not significantly differ between female and male groups. In contrast, ATHL exhibited significantly higher erythrocyte levels of Nrf2 than the SED group (*p* < 0.0001; Table 1S), as previously demonstrated (Daniele et al. 2018a, b, c, d). This evidence occurred independently from sex (erythrocyte levels in female ATHL vs female SED, *p* = 0.0056; erythrocyte levels in male ATHL vs male SED, *p* < 0.0001; Table 3).

Furthermore, to investigate additional mechanisms involved in oxidative stress, ND-related proteins, Nrf2 levels, the expression levels of selected circulating miRNAs, HDAC6, and DNMTs were determined in blood samples (in this sense, see discussion section). In particular, among the different miRNAs related to neurodegenerative processes, miR-153-3p was chosen because of its link with Nrf2 and the downstream genes, while miR-195-5p was selected for its ability to regulate Aβ transcriptional levels (Ai et al. 2013; J. Zhu et al. 2018). Herein, females and males presented comparable amounts of both miR-153 and miR-195 in plasma (miR-153, *p* = 0.4678; miR-195, *p* = 0.4137; Table 2). Conversely, both ATHL females (*p* = 0.0002; Table 3) and ATHL males (*p* = 0.0023; Table 3) showed higher plasma levels of miR-195 than the related SED groups. Of note, ATHL males showed reduced plasma levels of miR-153 (*p* = 0.0038; Table 3), while no significant differences were revealed in the female group (*p* = 0.1543; Table 3). Interestingly, although erythrocyte HDAC6 concentrations were comparable in males and females (*p* = 0.7061), they were significantly increased in ATHL compared to SED (*p* < 0.0001), considering both females (*p* < 0.0001; Table 3) and males (*p* < 0.0001; Table 3).

Finally, no significant differences in the plasma levels of DNMT (DNMT1 and DNMT3A) were evidenced among the examined groups.

### Correlations Between Clinical and Biochemical Parameters

All clinical and biochemical parameters were correlated by simple regression analysis, as summarized in Supplementary Table 1. Of note, the results below, depicted as scatter diagrams in Figs, 1,2, 3, 4, and 5, concern the correlations statistically significant, only, and report the simple correlation coefficient (“*R*2”). Weak correlations may indicate that other unmeasured factors likely contribute substantially to the variance in the respective parameters.

**Fig. 1:**
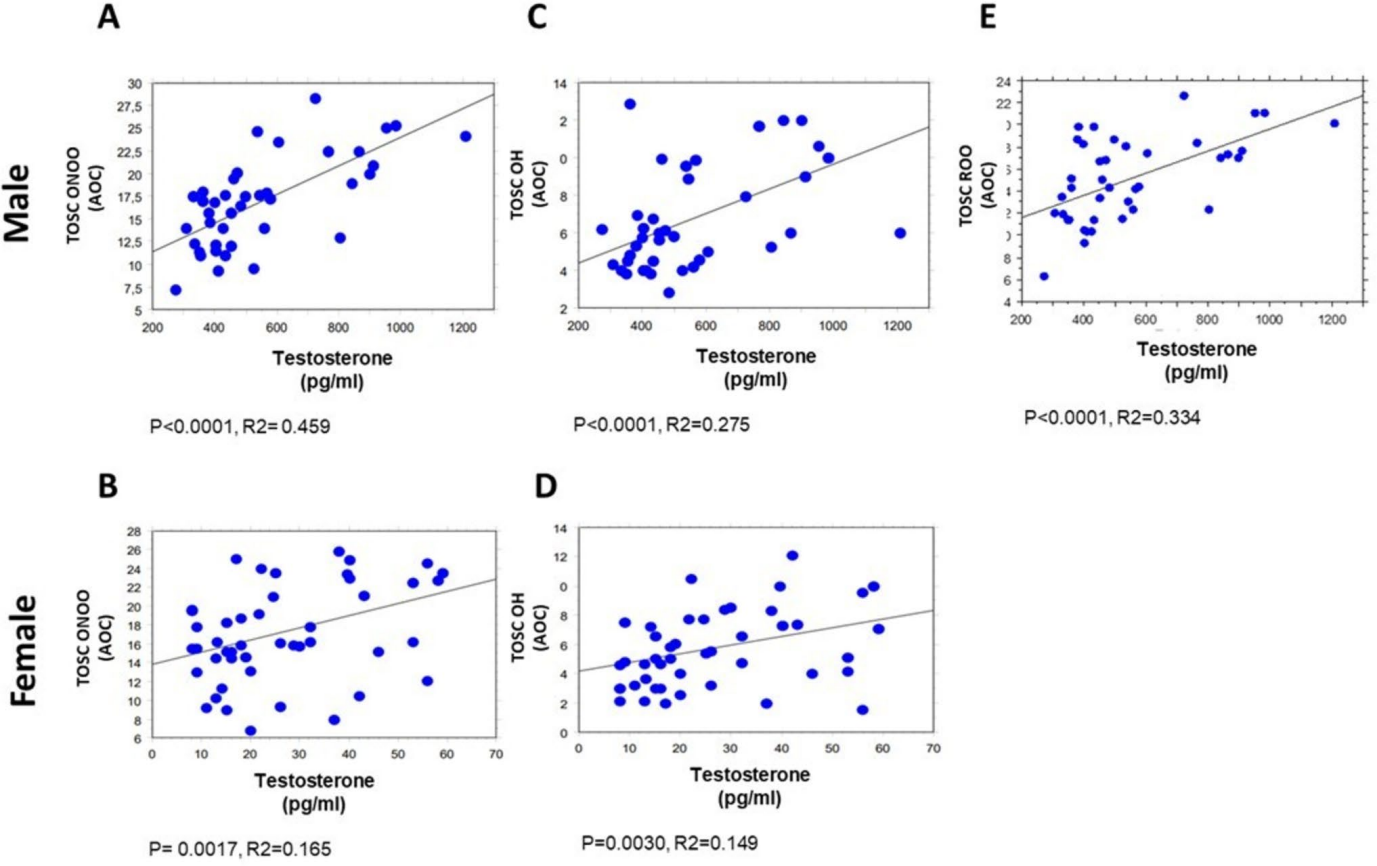
Correlations between testosterone and TOSC values in male (60 subjects) and female (60 subjects) groups. Correlation analysis between testosterone and TOSC values against peroxynitrite radicals (ONOO) (**A** and **B**), hydroxyl radicals (**C** and **D**), and peroxyl radicals (**E**). All correlations between the selected variables have been performed by simple linear regression analysis, using the StatView program (Abacus Concepts, Inc., SAS Institute, Cary, NC, United States). *p*- and *R*2 values obtained for each correlation are reported in the respective panel. TOSC ONOO, TOSC values vs peroxynitrite radicals (AOC); TOSC OH, TOSC values vs hydroxyl radicals (AOC); TOSC ROO, TOSC values vs peroxyl radicals (AOC)

Positive correlations were observed between testosterone levels and TOSC values vs peroxynitrite (*p* < 0.0001, *R*2 = 0.459 in males, Fig. 1A; *p* = 0.0017, *R*2 = 0.165 in females, Fig. 1B) and hydroxyl radicals (*p* < 0.0001, *R*2 = 0.275 in males, Fig. 1C; *p* = 0.0030, *R*2 = 0.149 in females, Fig. 1D). Moreover, a positive correlation between testosterone and TOSC vs peroxyl radicals was found in males (*p* < 0.0001, *R*2 = 0.334, Fig. 1E); conversely, this correlation was not reported in females (*p* ≥ 0.05).

**Fig. 2:**
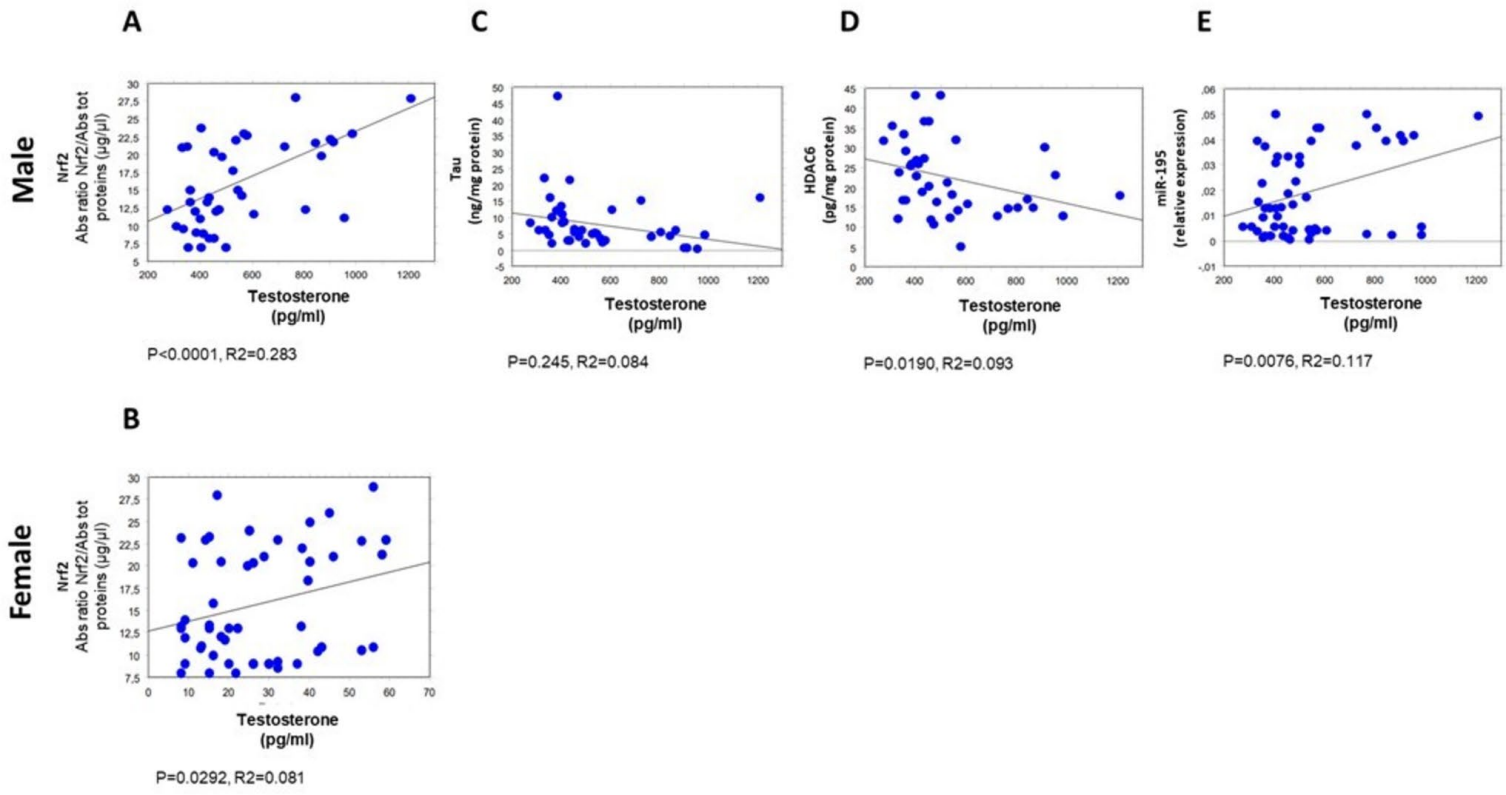
Correlations between testosterone and selected biochemical parameters in male (60 subjects) and female (60 subjects) groups. Correlation analysis of testosterone with Nrf2 (**A** and **B**), tau protein (**C**), HDAC6 (**D**), and miR-195 (**E**). All correlations between selected variables have been performed by simple linear regression analysis, using the StatView program (Abacus Concepts, Inc., SAS Institute, Cary, NC, United States). *p*- and *R*2 values obtained for each correlation are reported in the respective panel. Nrf2 nuclear factor erythroid 2-related factor 2, HDAC6 histone deacetylase 6, miR miRNA

Furthermore, a positive correlation was found between plasma testosterone and the erythrocyte levels of Nrf2 in both female and male subgroups (*p* < 0.0001, *R*2 = 0.283 in males, Fig. 2A; *p* = 0.0292, *R*2 = 0.081 in females, Fig. 2B). Of note, in the male subgroup only, an inverse correlation was evidenced between plasma testosterone concentration and erythrocytes levels of tau (*p* = 0.245, *R*2 = 0.084, Fig. 2C) or HDAC6 (*p* = 0.0190, *R*2 = 0.093; Fig. 2D); in contrast, a direct correlation was evidenced between plasma testosterone concentration and plasma levels of miR-195 (*p* = 0.0076, *R*2 = 0.117; Fig. 2E).

**Fig. 3:**
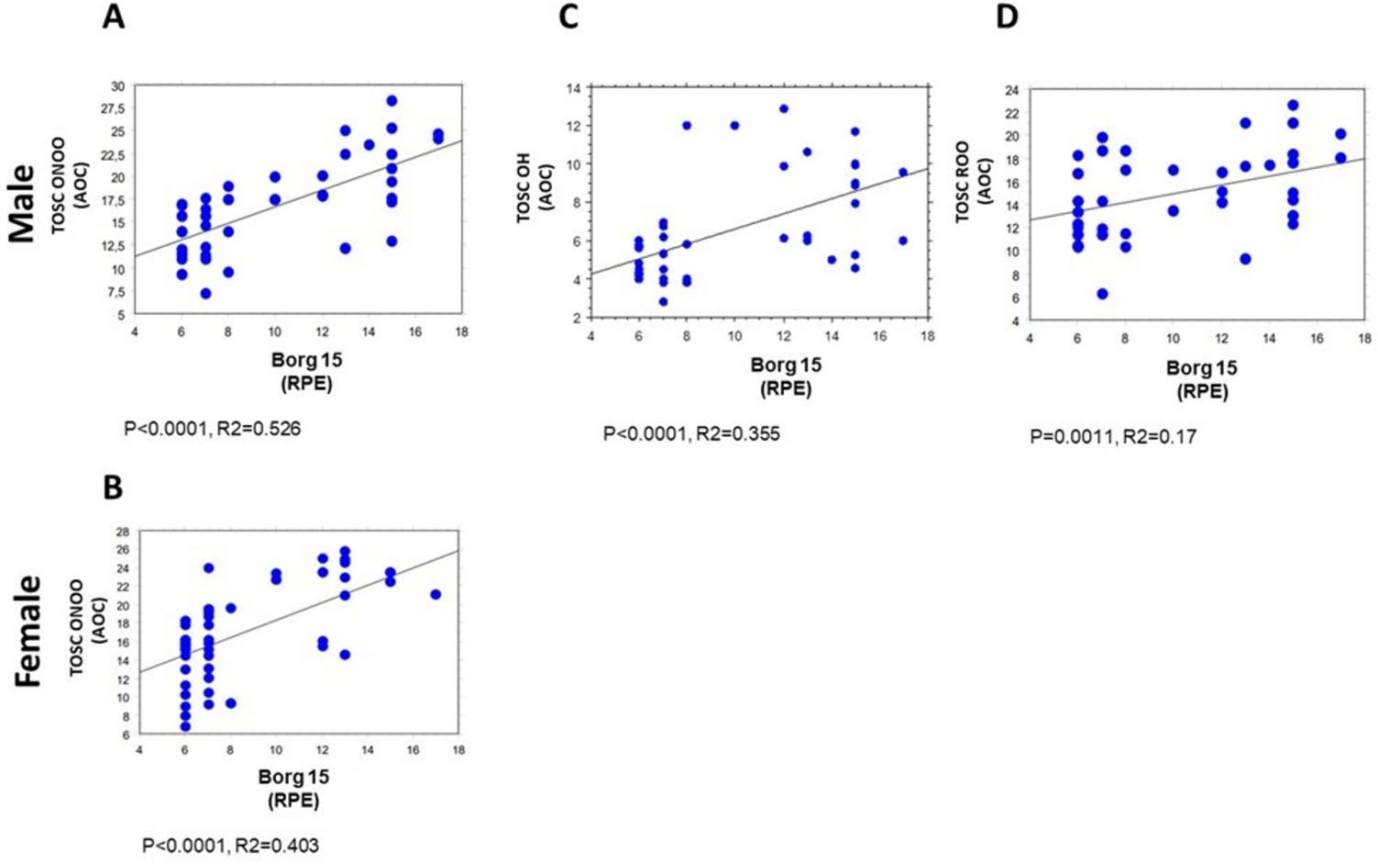
Correlations between Borg 15 and TOSC values in male (60 subjects) and female (60 subjects) groups. Correlations between Borg 15 and TOSC values in male and female subjects. Correlation analysis between Borg 15 and TOSC values against peroxynitrite radicals (ONOO) (**A** and **B**), hydroxyl radicals (**C**), and peroxyl radicals (**D**). All correlations between the selected variables have been performed by simple linear regression analysis, using the StatView program (Abacus Concepts, Inc., SAS Institute, Cary, NC, United States). *p*- and *R*2 values obtained for each correlation are reported in the respective panel. TOSC ONOO, TOSC values vs peroxynitrite radicals (AOC); TOSC OH, TOSC values vs hydroxyl radicals (AOC); TOSC ROO, TOSC values vs peroxyl radicals (AOC)

As expected, a positive correlation was noticed between the Borg 15 scale and plasma TOSC values vs peroxynitrite (*p* < 0.0001, *R*2 = 0.526, Fig. 3A), peroxyl (*p* = 0.0011, *R*2 = 0.17, Fig. 3C), and hydroxyl radicals (*p* < 0.0001, *R*2 = 0.355, Fig. 3D) in males. Of note, when the female group was considered, a significant direct correlation was noticed between the Borg 15 scale and plasma TOSC values versus peroxynitrite radicals only (*p* < 0.0001, *R*2 = 0.403, Fig. 3B).

**Fig. 4:**
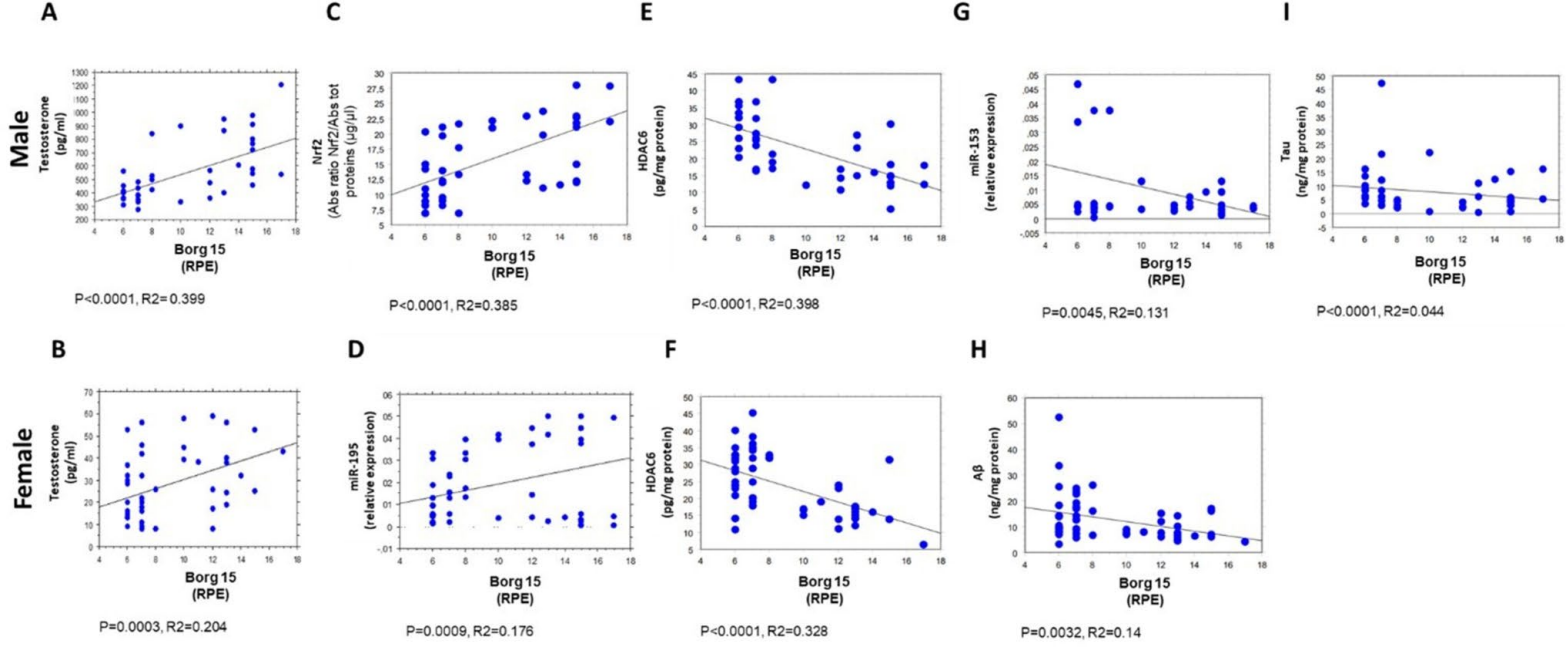
Correlations between Borg 15 and selected biochemical parameters in male (60 subjects) and female (60 subjects) groups. Correlations between Borg 15 and testosterone (**A** and **B**) and Borg 15 and HDAC6 (**E** and **F**) in females and males; correlations considering Borg 15 versus Nrf2 (**C**), miR-153 (**G**), and Tau (**I**) in males as well as versus miR-195 (**D**) and Aβ (**H**) in females. All correlations between the selected variables have been performed by simple linear regression analysis, using the StatView program (Abacus Concepts, Inc., SAS Institute, Cary, NC, United States). *p*- and *R*2 values obtained for each correlation are reported in the respective panel. Borg 15, Borg rating of perceived exertion scale (RPE); miR-195, miRNA 195; Nrf2, nuclear factor erythroid 2-related factor 2; HDAC6, histone deacetylase 6; miR-153, miRNA 153; Aβ, β-amyloid; Tau

**Fig. 5:**
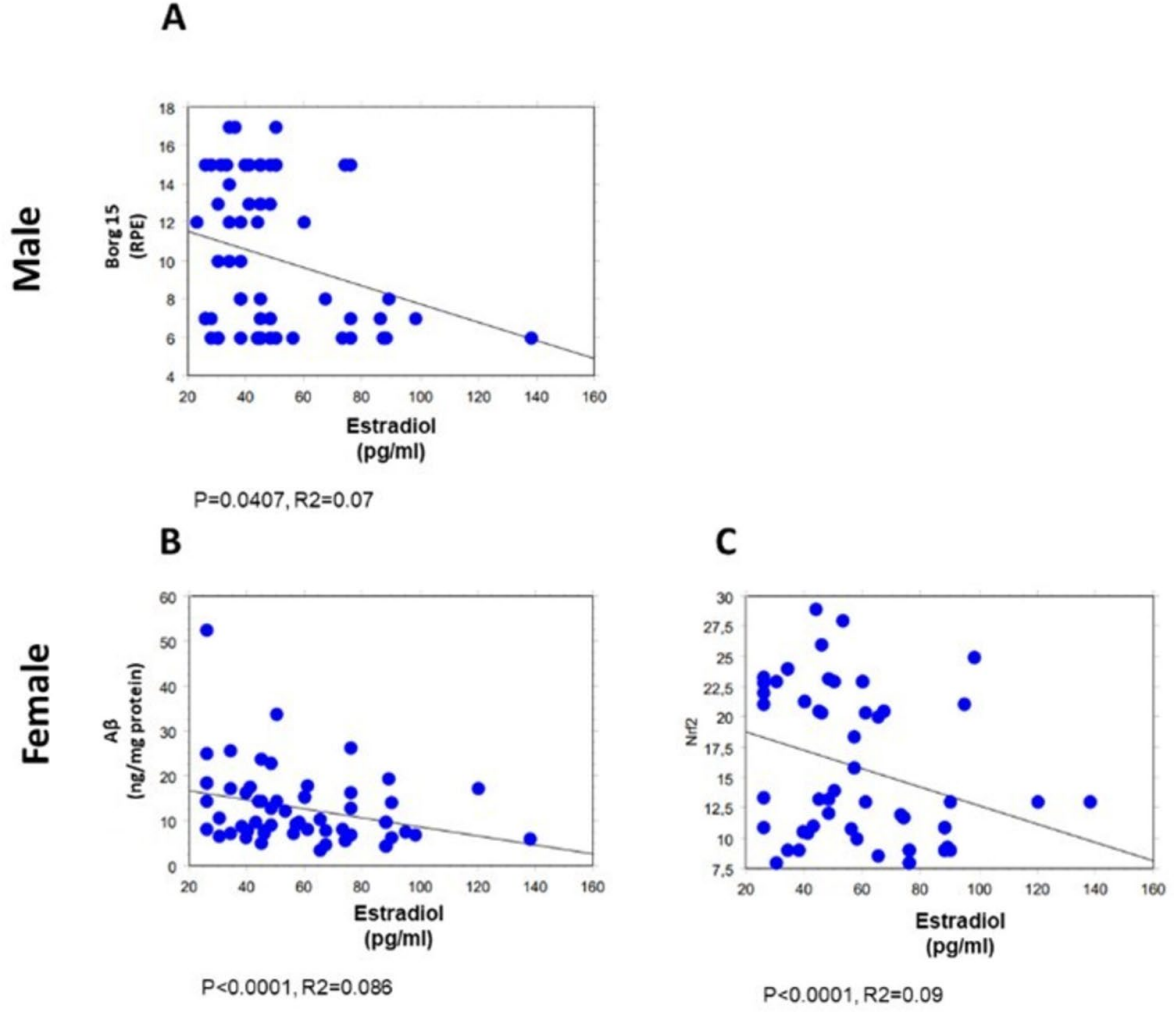
Correlations between estradiol and selected biochemical parameters in male (60 subjects) and female (60 subjects) groups. Correlation analysis between estradiol and Borg 15 (**A**), Aβ (**B**), and Nrf2 (**C**). All correlations between the selected variables have been performed by simple linear regression analysis, using the StatView program (Abacus Concepts, Inc., SAS Institute, Cary, NC, United States). *p*- and *R*2 values obtained for each correlation are reported in the respective panel

Moreover, both in males and in females, a significant direct correlation was noticed between Borg15 and plasma levels of testosterone (*p* < 0.0001, *R*2 = 0.399 in blood samples of males, Fig. 4A; *p* = 0.0003, *R*2 = 0.204 in blood samples of females, Fig. 4B). Consistently, an inverse correlation was evidenced between plasma estradiol concentration and the Borg15 in the male group (*p* = 0.0407, *R*2 = 0.07, Fig. 5A).

Interestingly, a significant positive correlation was observed between Borg15 and Nrf2 in males (*p* < 0.0001, *R*2 = 0.385, Fig. 4C), and miR-195 in females (*p* = 0,0009, *R*2 = 0.176, Fig. 4D). Furthermore, a significant inverse correlation was evidenced between Borg 15 and the erythrocyte concentration of HDAC6 (*p* < 0.0001, *R*2 = 0.398 in males, Fig. 4E; *p* < 0,0001, *R*2 = 0.328 in females, Fig. 4F), miR-153 (*p* = 0.0045, *R*2 = 0.131 in males, Fig. 4G), and erythrocyte tau levels (*p* < 0.0001; *R*2 = 0.044 in males, Fig. 4I).

Finally, it is interesting to note the significant inverse correlation between physical activity level and the erythrocyte concentration of Aβ (*p* = 0.0032, *R*2 = 0.14, Fig. 4H) in females only; consistently, a significant negative correlation was observed between plasma estradiol concentrations and erythrocyte Aβ (*p* < 0.0001, *R*2 = 0.086, Fig. 5B), as well as erythrocyte Nrf2 levels (*p* < 0.001, *R*2 = 0.09, Fig. 5C) in the same group.

## Discussion

Cerebral adaptations to long-term exercise contribute to counteracting the negative effects of aging and neurodegenerative phenomena, although sex greatly impacts brain responses to regular physical activity (Barha & Liu-Ambrose 2018). In the present study, sex differences in exercise-modulated molecular and epigenetic mechanisms were investigated in a population of physically active subjects, compared to healthy sedentary controls. The clinical and biochemical parameters have highlighted the main following results: (i) females presented higher β-amyloid and a-synuclein levels than males; (ii) in females, β-amyloid levels were reduced with regular physical activity and inversely related to estradiol concentrations, and in males, tau concentrations decreased with regular physical activity and were inversely related to testosterone levels; (iii) despite males and females presented a comparable plasma AOC, in the female subgroup, regular PA was not able to induce an enhancement of plasma AOC versus peroxyl and hydroxyl radicals but rather versus peroxynitrite species only. Furthermore, we found that regular physical activity (i) increased Nrf2 and miR-195 levels and (ii) decreased HDAC6 and miR-153 concentrations, thereby promoting antioxidant and autophagic processes that seem to be protective mechanisms against neurodegeneration. Overall, the current study provides evidence about the gender influence on blood accumulation of ND proteins as well as the ND-related pathological mechanisms’ modulation in relation to regular physical activity, especially as concern molecular mechanisms of antioxidant responses.

Physical activity is known to induce several positive health effects, improving cardiovascular functions, neuroplasticity, and also age-related disorders, such as cognitive decline (Daniele et al. 2020; Hötting & Röder 2013; Wu et al. 2019). Interestingly, some evidence has indicated different responses, depending on the gender, to physical activity, which is an effective physiological stimulus, in terms of heart rate variability (Koenig & Thayer 2016; Spina et al. 2019), muscle activation (Alizadeh et al. 2020), body composition (Sawada et al. 2021), and metabolic processes (Tarnopolsky 2008). Nevertheless, there is still a lack of investigations concerning the molecular mechanisms of sex-related differences in physical activity.

Concerning animal experiments, most of the studies have examined the acute effects of exercise in young or adolescent animals. Aerobic training has been reported to improve learning and memory in a greater way in males on conditioned-avoidance and non-spatial memory tasks. However, this typical advantage of young males for spatial memory performance has been not observed in aged subjects (Bowman et al. 2006), suggesting that aging is associated with changes in the pattern of sex differences. In contrast, voluntary exercise in rats has been demonstrated to increase the levels of brain-derived neurotrophic factor compared to controls, with larger effects in females (Titterness et al. 2011). Accordingly, in a mouse model of AD, exercise has proven to enhance hippocampal-dependent reference memory to a greater extent in young adult female mice compared with male mice (Pietropaolo et al. 2008). Overall, literature data suggest that sex is an important factor to consider, and studies directly assessing sex differences in the ability of regular chronic exercise to improve brain function are needed (Barha et al. 2017)*.*

Herein, a cohort of healthy adult subjects of both sexes, divided into athletes (physically active individuals, ATHL) and age- and sex-matched sedentary controls (SED), was enrolled to study sex-related differences in antioxidant capacity and proteasome regulation. In our hand, physical activity was confirmed to enhance plasma AOC, as previously reported (Scarfò et al. 2021, 2022). Nevertheless, although no differences in plasma AOC between males and females were revealed, the physical activity-induced increase in TOSC values was less pronounced in the female group, in which no significant correlation between antioxidant capability toward hydroxyl and peroxyl radicals, and the levels of physical activity have been highlighted. On the contrary, in the male group, the AOC versus all radicals was positively related to both physical activity and testosterone. These data may be ascribed to an influence of the hormonal asset on the biological response to physical activity practice. Several studies have examined the sex-specific mechanisms of action of testosterone and redox balance (Cruz-Topete et al. 2020), evidencing a clear association of total antioxidant capacity with sex hormones (Demirbag et al. 2005). Consistent with our data, a strong correlation between plasma AOC and testosterone has been found in men, and testosterone has been shown to enhance plasma antioxidant enzymes in patients with hypogonadism (Demirbag et al. 2005).

Of note, herein ATHL exhibited higher testosterone levels compared to SED, both in male and female groups. We can speculate that regular exercise positively affects testosterone production, as proven by the positive correlation between the Borg 15 scale and testosterone levels, independently from the sex. In this sense, it has been largely demonstrated that serum testosterone levels seem to benefit from regular physical activity (Cumming et al. 1986; Rosety et al. 2017) although several factors could influence its plasma concentrations including the type and the intensity of exercise, and the interindividual variability (Riachy et al. 2020). Consistent with our data, several studies have suggested the strict relationship between regular exercise and increased circulating testosterone levels in physically active individuals (D’Andrea et al. 2020), particularly in males. Future studies should focus on clarifying the metabolic and molecular mechanisms whereby exercise may affect serum testosterone concentrations in the short and long-terms, and furthermore, how this affects downstream mechanisms.

Moreover, a negative correlation between the Borg 15 scale and estradiol levels was evidenced, with a possible negative and less effective antioxidant capacity. Consistently, previous studies have shown that physical activity can decrease circulating levels of estradiol (Jasienska et al. 2006).

Then, the blood concentrations of neurodegeneration-related proteins, i.e., Aβ, α-syn, and tau were investigated (Baldacci et al. 2019; Daniele et al. 2021). In particular, erythrocytes only were chosen to investigate the accumulation of ND-related protein, as a good peripheral model to study biochemical alteration related to aging and oxidative stress processes (Kiko et al. 2012; Orrico et al. 2023), accumulating Aβ (Kiko et al. 2012) and representing the major source of α-syn (Barbour et al. 2008). Males presented significantly lower levels of Aβ and total α-syn than females. Consistently, the Aβ level has been demonstrated to be higher in females than in males both in animals of the same age (Carroll et al. 2010; J. Wang et al. 2003) and in human healthy subjects (Prete et al. 2020). Of note, herein we examined the 1–42 peptide of Aβ, which is the most predominant isoform in the amyloid fibrils (Upadhyay et al. 2023) and whose accumulation has been associated mainly with AD development (El‐Agnaf et al., 2006).

In contrast, a previous study has reported no significant differences in erythrocyte accumulation of total α-syn between females and males, but higher concentrations of α-syn/tau heterocomplexes as compared to age-matched men (Prete et al. 2020). Nevertheless, the examination of ND-related proteins and their different oligomeric isoform in other compartments than erythrocytes would give a broader vision. Overall, our preliminary findings highlight that the above-mentioned differences may be mediated by organizational actions of sex steroid hormones during development. In this sense, the over-representation of AD in females is well-documented, together with a higher propensity to Aβ plaques, and widespread tau pathology (Hugenschmidt et al. 2021).

Consistent with these findings, in our study, estradiol correlated negatively to Aβ concentration in the female group, according to another study in which it has been shown that a decrease in estradiol levels in ovariectomized rats led to increased serum levels of Aβ (Kridawati et al. 2020)*.* From a mechanistic point of view, estrogen may exert beneficial effects by downregulating BACE1 protein expression through direct binding and complex formation with estrogen response elements and cofactors, as suggested in mixed neuronal/glial cultures (Cui et al. 2022).

Moreover, regular physical activity decreased blood levels of Aβ and tau, consistent with the beneficial effects of regular physical activity in cognitive maintenance, Aβ turnover modulation, and brain redox status improvement. Particularly, physical activity was able to decrease Aβ levels in females and tau in males. These findings are of particular interest considering that Aβ and tau are the pivotal pathological proteins of Alzheimer’s and Parkinson’s disease, respectively. Thus, considering that Alzheimer’s disease is more frequent in females and Parkinson’s disease is overrepresented in males, it can be speculated that regular physical activity exerts sex-related neuroprotective effects (Cerri et al. 2019; Fernández et al. 2023).

Then, the molecular mechanisms potentially related to ND-related proteins and oxidative stress were investigated. First, the levels of Nrf2, a transcription factor that positively regulates the expression and activity of cytoprotective genes during periods of oxidative stress, were assessed. No significant differences were evidenced among males and females, although some reports suggested an augmentation of Nrf2 expression and activity in female tissues through an estrogen-dependent mechanism. Of note, these studies have evidenced an Nrf2 activation in specific cellular models that occur in stressful conditions as well as in response to specific cellular cues (De La Rosa et al. 2020). In contrast, estradiol was negatively related to Nrf2 in females in the present work, consistently with an in vivo study, in which the decrease of Nrf2 protein has been observed in mice treated with 17β-estradiol (E_2_) (Sobočanec et al. 2015). Nevertheless, in interpreting these results, compensatory or secondary mechanisms related to the rapid Nrf2 activation by estrogens cannot be excluded. The interaction between Nfr2 and estrogens may lead to metabolic reprogramming through upregulation of enzymes for glucose metabolism and at the same time suppresses generation of ROS to protect cells from oxidative damages.

Consistent with the abovementioned association, herein we noticed a positive association between Nrf2 levels and testosterone concentrations, in line with the greater antioxidant capability evidenced in subjects with higher testosterone levels. Moreover, physical activity induced a significant increase in Nrf2 concentrations, coherent with the previous reports (Piccarducci et al. 2021). To deepen the epigenetic factors around Nrf2, the concentration of HDAC6 and two specific miRNAs were investigated. In particular, we investigated the blood concentrations of (i) HDAC6, which has been proven to target the transcription factor Nrf2, enhancing its binding to antioxidant response elements (B. Wang et al. 2012); (ii) miR-195, which has been demonstrated to downregulate Aβ production (H.-C. Zhu et al. 2012) and to exert neuroprotective effects (Ai et al. 2013); and (iii) miR-153 has been proven to be linked to Keap1-Nrf2 axis and the downstream genes (Liu et al. 2018; Prasad 2017) and to inhibit autophagy (Zou et al. 2016). In our hands, HDAC6 concentrations were independent of the sex but were reduced by regular physical activity. Since HDAC6 can inhibit the Nrf2 expression and bind to antioxidant response elements (Piccarducci et al. 2021; Singh 2018), these data are consistent with the comparable levels of Nrf2 and with the enhancement of the latter in athletes.

Finally, herein we proved that females and males exhibited similar expression of miR-153 and miR-195. Interestingly, the level of physical activity was inversely related to miR-153 one, as previously demonstrated in human subjects (Piccarducci et al. 2021). These data are consistent with literature reports demonstrating that miR-153 enhances oxidative stress by negatively regulating Nrf2: in this sense, regular physical activity is able to activate Nrf2 and inhibit miR-153 expression, thereby enhancing antioxidant responses. On the other hand, physical activity was related directly to miR-195 expression, coherent with the Aβ decrease presented by active subjects: indeed, miR-195 has been proven to downregulate the level of Aβ by inhibiting the translation of beta-site APP cleaving enzyme 1 (BACE1) (H.-C. Zhu et al. 2012).

## Conclusions

Herein, we showed that females and males have different levels of ND-related proteins in blood and that physical activity differentially affects the levels of these proteins as well as plasma antioxidant capability depending on gender. We acknowledge the following limitations. First, we conducted an observational study, in which the athletes were not trained by our staff directly; participants were healthy community-dwelling volunteers so we cannot exclude volunteer bias. Furthermore, in interpreting our results, it should be mentioned that the complexity of the hormonal axis, even considering a strict range of age, may render it difficult to generalize the obtained data. Animal studies will be planned to illustrate the causal relationship between physical activity and hormone changes in different sexes in a more controlled manner. Finally, the mean age of the enrolled subject, with the aim of considering pre-menopausal women, does not allow the effective evaluation of neuroprotective effects in aged or middle-aged brains.

Surely, regular physical activity practice is a promising strategy to counteract the deleterious effects of aging and neurodegeneration (Barha & Liu-Ambrose 2018). Male or female gender can represent an important factor in modulating the relationship between exercise and neuroprotection.

Future analyses to gap the limitation of the study could consider investigating the potential sex difference in exercise efficacy could lead to more effective exercise-based interventions to promote brain aging in healthy individuals, as well as in patients with neurodegenerative diseases.

## Supplementary Information

Below is the link to the electronic supplementary material.

Supplementary file1 (DOCX 19 KB)

## Data Availability

Data is provided within the manuscript or supplementary information files.
