# Supplementary material for: Sex Differences in Blood Accumulation of Neurodegenerative-Related Proteins and Antioxidant Responses to Regular Physical Exercise
Source: J Mol Neurosci. 2024 Nov 5;74(4):105. doi: 10.1007/s12031-024-02278-4 (PMC11535074; doi:10.1007/s12031-024-02278-4)
Supplement: Supplementary file 1 — Supplementary file1 (DOCX 19 KB) [file 12031_2024_2278_MOESM1_ESM.docx]

**Supplementary Materials**

**Original Article**

**Sex differences in blood accumulation of neurodegenerative-related proteins and antioxidant responses to regular physical exercise**

Elisa Chelucci ^1*^, Giorgia Scarfò ^2*^, Rebecca Piccarducci ^1^, Antonio Rizza ^3^, Jonathan Fusi ^2^, Francesco Epifani ^4^, Sara Carpi ^5^, Beatrice Polini ^6^, Laura Betti ^1^, Barbara Costa ^1^, Sabrina Taliani ^1^, Vito Cela ^7^, Paolo Artini ^7^, Simona Daniele ^1, #^, Claudia Martini ^1^, Ferdinando Franzoni ^2^ **.**

^1^ Department of Pharmacy, University of Pisa, Italy, E.C., [e.chelucci@studenti.unipi.it](mailto:e.chelucci@studenti.unipi.it); R.P., [rebecca.piccarducci@unipi.it](mailto:rebecca.piccarducci@unipi.it); L.B., [laura.betti@unipi.it](mailto:laura.betti@unipi.it) ; B.C., [barbara.costa@unipi.it](mailto:barbara.costa@unipi.it) ; S.T., [sabrina.taliani@unipi.it](mailto:sabrina.taliani@unipi.it) ; C.M., [claudia.martini@unipi.it](mailto:claudia.martini@unipi.it);

^2^ Division of General Medicine, Department of Clinical and Experimental Medicine, University of Pisa, Italy, G. S., [g.scarfo1@studenti.unipi.it](mailto:g.scarfo1@studenti.unipi.it) ; J.F., [jonathan.fusi@gmail.com](mailto:jonathan.fusi@gmail.com) ; F.F., [ferdinando.franzoni@unipi.it](mailto:ferdinando.franzoni@unipi.it);

^3^ Interventional Cardiology Division, Gaetano Pasquinucci Heart Hospital, Fondazione Toscana Gabriele Monasterio, Massa, Italy, A. R., [antonio.rizza@ftgm.it](mailto:antonio.rizza@ftgm.it);

^4^ Department of Juridical and Economic Sciences, Pegaso Telematic University; Fanfani. Diagnostics and Health. Firenze, Italy, F. E. [francesco.epifani@istitutofanfani.it](mailto:francesco.epifani@istitutofanfani.it);

^5^ Department of Health Sciences, University “Magna Graecia” of Catanzaro, and National Enterprise for nanoScience and nanoTechnology (NEST), Istituto Nanoscienze-CNR and Scuola Normale Superiore, Pisa, Italy, S.C, sara.carpi@unicz.it

^6^Department of Pathology, University of Pisa, Italy, B.P., b.polini@studenti.unipi.it

^7^ Division of Gynecology and Obstetrics, Azienda Ospedaliero Universitaria Pisana and Department of Clinical and Experimental Medicine, University of Pisa, Italy, V.C., [celav2001@gmail.com](mailto:celav2001@gmail.com), P.A., [paolo.artini@unipi.it](mailto:paolo.artini@unipi.it)

*these authors equally contributed to the work.

# correspondence to: S.D., Department of Pharmacy, University of Pisa, Italy; simona.daniele[@unipi.it](mailto:rebecca.piccarducci@unipi.it).

| **Parameters** | **ATHL** | **SED** |
| --- | --- | --- |
| **TOSC ROO**  (AOC) | 16.44 ± 4.56ˆˆ | 14.42 ± 3.72 |
| **TOSC OH**  (AOC) | 7.43 ± 2.35ˆˆˆˆ | 5.49 ± 2.42 |
| **TOSC ONOO**  (AOC) | 20.93 ± 4.03ˆˆˆˆ | 14.75 ± 3.94 |
| **Aβ**  (ng/mg protein) | 9.31 ± 6.25ˆ | 12.52 ± 8.34 |
| **Tau**  (ng/mg protein) | 5.61 ± 3.54ˆˆ | 9.48 ± 8.89 |
| **α-syn**  (ng/mg protein) | 60.31 ± 56.59 | 59.65 ± 58.74 |
| **Oligomeric α-syn**  (ng/mg protein) | 10.02 ± 4.03 | 11.25 ± 5.60 |
| **Nrf2**  (Abs ratio Nrf2/Abs tot proteins (μg/μL) | 19.32 ± 5.82ˆˆˆˆ | 13.68 ± 5.48 |
| **miR-153**  **(**relative expression) | 0.005 ± 0.006ˆˆ | 0.013 ± 0.016 |
| **miR-195**  **(**relative expression) | 0.025 ± 0.019ˆˆˆ | 0.014 ± 0.013 |
| **HDAC6**  **(**pg/mg protein**)** | 16.30 ± 6.52ˆˆˆˆ | 27.11 ± 8.35 |
| **DNMT1**  (pg/mg protein) | 448.35 ± 355.51 | 431.13 ± 268.44 |
| **DNMT3A**  (pg/mg protein) | 141.74 ± 111.64 | 167.78 ± 102.32 |

**Supplementary Table 1S.** Selected clinical and biochemical parameters for ATHL and SED groups. The reported parameters were compared between the whole group of ATHL and the whole group of SED subjects, independently from biological sex. Statistical analysis was performed by unpaired t-test between ATHL (60 subjects) and SED (60 subjects). All data are expressed as mean ± SD..ˆ p<0.05, ˆˆp≤0.01; ˆˆˆp<0.001 ˆˆˆˆp<0.0001, ATHL vs SED subjects. TOSC ROO: TOSC values vs peroxyl radicals; TOSC OH: TOSC values vs hydroxyl radicals; TOSC ONOO: TOSC values vs peroxynitrite radicals; Aβ: beta-Amyloid; α-syn: alpha-synuclein; Nrf2: nuclear factor erythroid 2-related factor 2; miR: miRNA; HDAC6: histone deacetylase 6; DNMT1: DNA methyltransferase 1; DNMT3A: DNA methyltransferase 3A
